# Case report: full recovery from dialysis-requiring renal failure after surgical repair of a completely occluded renal artery in a patient with a single kidney

**DOI:** 10.1186/s12882-025-04352-4

**Published:** 2025-08-01

**Authors:** Marta Kantauskaite, Klaus Grabitz, Lars Christian Rump, Sebastian Alexander Potthoff

**Affiliations:** 1 Department of Nephrology, Heinrich-Heine University, Medical Faculty, University Clinic Duesseldorf, Moorenstr. 5, 40225 Duesseldorf, Germany; 2 Department of Vascular and Endovascular Surgery, Heinrich-Heine University, Medical Faculty, University Clinic Duesseldorf, Duesseldorf, Germany

**Keywords:** Renal failure, Renal infarction, Renal artery thrombosis, Aortic-renal bypass, Collateral vasculature

## Abstract

**Introduction:**

Renal infarction is an extremely rare condition occurring in the context of structural or functional cardiac abnormalities, renal artery injury or coagulative syndromes. Although the clinical presentation of renal infarction is often nonspecific, the presence of symptoms such as back pain, high blood pressure, nausea and fever should raise suspicion, particularly in the emergency setting. Timely diagnosis is crucial for preserving renal function, whether through minimally invasive procedures or bypass surgery.

**Case presentation:**

We present a case of a young male who developed acute occlusion of an aortic-renal bypass supplying a solitary left kidney. The patient exhibited resistant arterial hypertension and acute oligo-anuric kidney injury requiring dialysis. Despite the total occlusion of the aorto-renal bypass on imaging, doppler ultrasound demonstrated moderate renal tissue perfusion, likely maintained via collateral vasculature. The existence of a previous prolonged ischemic condition may have led to the formation of collateral-dependent circulation. While insufficient for pressure-dependent diuresis, the collateral flow preserved renal tissue oxygenation.

**Conclusions:**

Collateral perfusion should be evaluated in cases of renal infarction, particularly when the main renal artery is occluded. Adequate collateral circulation might preserve renal tissue viability beyond the typical ischemic window.

## Background

Acute renal infarction is an extremely rare but potentially devastating emergency. Partial or complete occlusion of the renal artery or its branches leads to reduced renal perfusion, resulting in ischemia and, ultimately, irreversible tissue necrosis. Depending on the size of the area with loss of perfusion, a wide variation of clinical symptoms has been reported. Most often patients report sudden flank or back pain, nausea, vomiting, fever, high blood pressure and/or reduced urine production [1, 2]. Nonspecific nature of these symptoms can delay diagnosis, often resulting in misidentification such as pyelonephritis or nephrolithiasis, thereby compromising outcomes. With a prevalence of approximately 1% and even lower incidence [1, 3], data on the treatment of acute renal infarction remain limited. Additionally, there are no clear clinical or biochemical predictors of treatment success. We report the case of a 41-year-old male patient presenting with acute oligo-anuric renal failure due to occlusion of an aortic-renal bypass supplying his solitary left kidney.

## Case presentation

A 41-year-old Caucasian male with a history of well-controlled arterial hypertension presented to the emergency department in poor general condition with complaints of nausea. His blood pressure was 210/100 mmHg, with a normal heart rate of 82 bpm. Further physical examination revealed no additional abnormalities, especially no signs of pulmonary or peripheral edema. The patient did not report any back or flank pain.

Laboratory results indicated acute renal injury: creatinine 14.3 mg/dl (1246.4 µmol/l) (baseline 1.1 mg/dl (97.26 µmol/l)) and an estimated glomerular filtration (eGFR) lower than 10 ml/min/1.73 m^2^. Urea levels were increased (149 mg/dl). Serum potassium was 4.6 mmol/l and a mild metabolic acidosis (pH 7.33, bicarbonate 17.2 mmol/l, base excess − 9.0 mmol/l) was present. Normocytic, normochromic anemia with a hemoglobin value of 9.8 g/dl was noted, likely due to renal failure as a reduced reticulocyte production index of 0.3 with no sign of inflammation was observed. At the presentation, the patient was oligo-anuric. Urinalysis showed no leukocytes or erythrocytes and proteinuria of 228.6 mg/gC. Lactate dehydrogenase was within normal range (135 U/l).

Previous medical conditions included the presence of arterial hypertension, which was managed with triple therapy. Furthermore, at the age of 10 years, he underwent right nephrectomy due to an atrophic right kidney. Later in life, at the age of 20, he was hospitalized with nausea, vomiting, and severe hypertension (230/100 mmHg). At that time, he suffered from acute kidney injury with serum creatinine of 11.6 mg/dl (1125.67 µmol/l), eGFR 6 ml/min/1.73 m^2^ and normal urine output. The cause of acute kidney injury was left renal artery thrombosis. Unfortunately, thrombectomy was unsuccessful, so an aortic renal bypass using an autologous vein graft was performed. After the surgery, renal function fully recovered with a serum creatinine of 1.0 mg/dl (88.4 µmol/l), eGFR 110 ml/min/1.73 m^2^. Since then, the patient remained asymptomatic and has been regularly seen by a doctor.

**Fig. 1:**
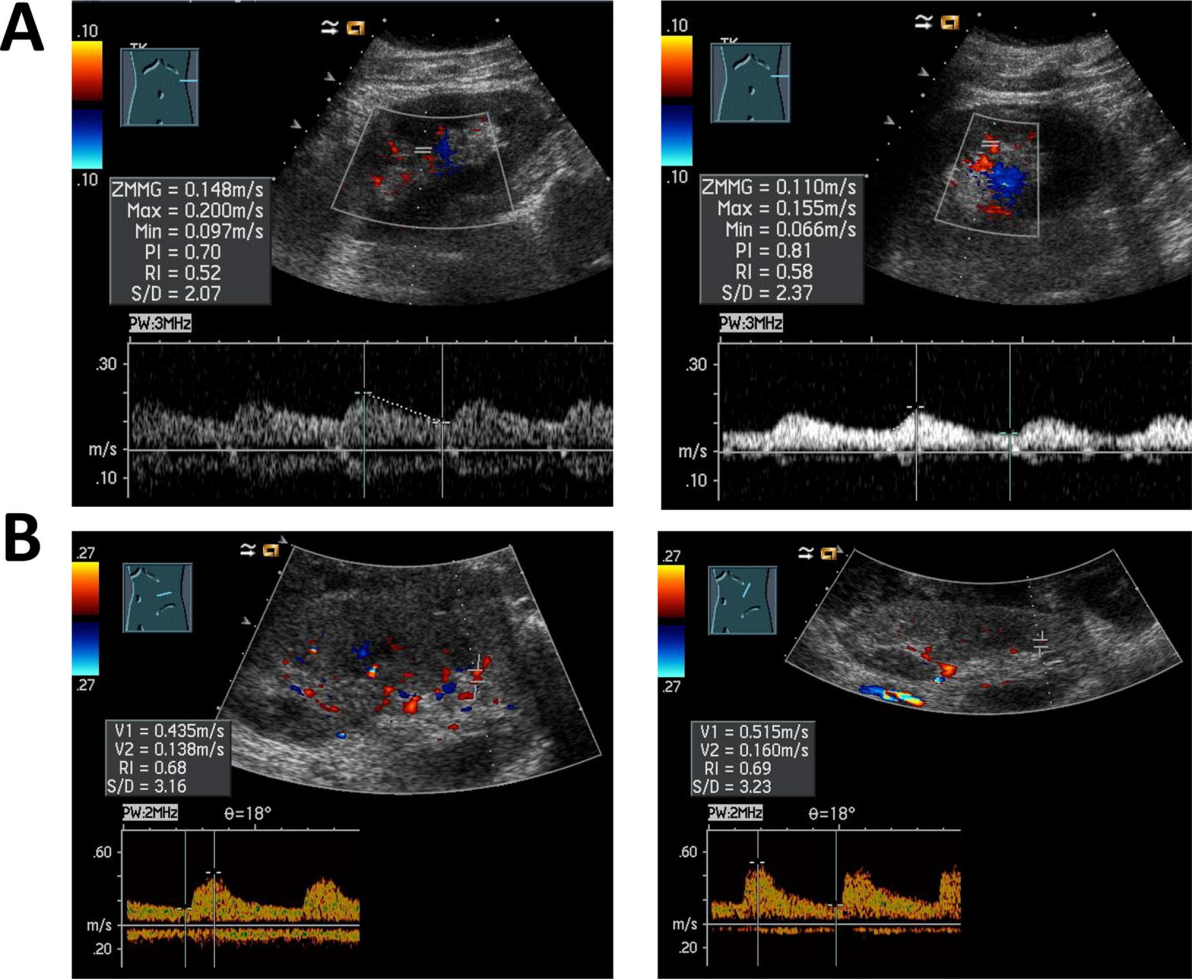
Renal doppler ultrasound before and after treatment for acute left aorta-renal bypass occlusion. **A** – both images represent the renal doppler ultrasound taken during the patient’s presentation. The left renal artery was not identifiable; however, a post-stenotic waveform was detected in renal parenchyma. Resistive indices (RI) ranged from 0.52 to 0.58, which is below that is expected for the 40-year-old male with a history of arterial hypertension. Additionally, prolonged acceleration time in Doppler waveforms was identified. **B** – after the graft placement between the aorta and the renal artery, doppler ultrasound showed an improved perfusion profile of the left kidney. RI´s exhibited a systolic peak, ranging from 0.64 to 0.66. Moreover, blood flow in the renal artery was measured with a maximal value of 195 cm/s

**Fig. 2:**
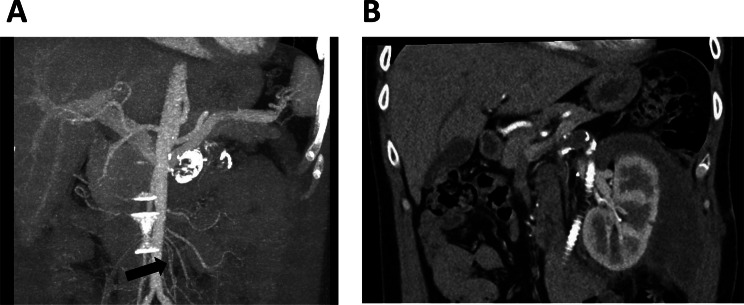
CT angiography of the abdominal aorta and its branches. **A** – Occlusion of the left aortal-renal bypass with calcification of the vein graft. Additionally, well-developed collateral circulation is indicated (black arrow). **B** – Recovery of perfusion in the left kidney following the placement of aorto-renal ePTFE (expanded polytetrafluoroethylene) vascular graft

Based on the patient’s medical history, an acute occlusion of the graft was suspected. Further diagnostic evaluation included a renal ultrasound, which showed a normal-sized left kidney measuring 12 cm, with no parenchymal changes or hydronephrosis. The Doppler ultrasound revealed post-stenotic waveforms, known as “tardus parvus”, and low resistive indices (RI) ranging from 0.52–0.58 (as shown in Fig. 1A), which were significantly lower than expected for the patient’s age. CT angiography confirmed a total occlusion of the left renal artery graft, likely due to thrombosis. Additionally, well-developed collateral vasculature was noted, as illustrated in Fig. 2A. Given the patient’s history, echocardiography was performed to exclude cardioembolic sources of renal graft thrombosis. The left ventricle and left atrium were normal in size, measuring 50 mm and 37 mm, respectively, with no evidence of interventricular septal hypertrophy. The left ventricular ejection fraction was 66% assessed by Simpson method, consistent with normal systolic function. No functional abnormalities of heart valves were identified.

At that time, we initiated dialysis treatment and intensified antihypertensive therapy. After evaluation of possible treatment options with the department of vascular and endovascular surgery, the patient was referred to aorto-renal bypass surgery. A 6 mm ePTFE (expanded polytetrafluoroethylene) vascular graft was used for the bypass. Postoperative recovery was uneventful. Diuresis resumed immediately, and serum creatinine declined to 1.2 mg/dl (106.1 µmol/l) with an eGFR of 73 ml/min/1.73 m^2^ within 6 days with no need of further dialysis. Doppler ultrasound showed improved intrarenal waveforms with early systolic peak and increased RIs of approximately 0.66 – well within the normal range for the patients’ age (as shown in Fig. 1B). Furthermore, CT angiography confirmed the improvement in renal perfusion, as illustrated in Fig. 2B. Other potential causes of acute renal infarction, such as coagulation or inflammatory disorders, have been excluded. Initially, the patient was set on anticoagulation therapy with rivaroxaban for 6 months. Due to good tolerability and given his previous medical history the decision for a lifelong anticoagulation therapy was made. Antihypertensive treatment was successfully reduced to 3 antihypertensive drugs while maintaining normotension.

## Discussion

Acute renal infarction is an extremely rare condition with a reported incidence below 1 % [1]. It results from the occlusion of the main renal artery or its branches, leading to decreased renal perfusion, oxygen deficiency, and ultimately tissue necrosis. Acute renal ischemia might induce sudden and sometimes severe pain in the back or flank area [3, 4]. The pain can be accompanied by other unspecific symptoms such as fever, nausea, vomiting and higher blood pressure [5–7]. In some cases, especially those with involvement of the main renal artery, reduced diuresis can be observed. Laboratory tests often show leukocytosis, increased levels of LDH and hematuria in urine samples [1, 4, 8]. Therefore, the clinical presentation of renal infarction is often misleading and other acute pathologies, such as pyelonephritis or renal colic might be diagnosed instead [9]. Patients frequently present without symptoms, with the diagnosis often being made incidentally during the evaluation of unrelated medical conditions [10]. Thus, acute renal infarction is probably an underdiagnosed clinical entity.

The causes of acute renal infarction can be divided into several groups. Most often, thromboembolism due to cardiac conditions, such as atrial fibrillation or endocarditis, is responsible for acute occlusion of the renal artery [1, 4, 8]. Other conditions leading to renal infarction are related to structural changes of the artery itself, as in cases of renal artery dissection and renal artery stenosis (RAS) due to atherosclerosis or fibromuscular dysplasia (FMD) [10]. In addition, coagulation and inflammatory disorders can be responsible for acute renal infarction as well. However, in approximately 30% of cases, the precise cause remains unclear and is classified as idiopathic renal infarction [1]. Our patient has suffered from two infarction episodes without evidence of cardio-embolic events, hypercoagulability, or inflammatory conditions. Given the young patient’s age and the history of an atrophic right kidney which was surgically removed in childhood, fibromuscular dysplasia (FMD) can be suspected. In fact, acute renal infarction can be the first and devastating clinical symptom of FMD, which occurs in approx. 1 % of cases [11, 12]. FMD is a non-atherogenic and non-inflammatory disease affecting mainly renal, vertebral and carotid arteries [13]. The involvement of renal arteries in the presence of FMD is seen in up to 90 % of cases [14–17]. Females are predominantly affected by FMD. Males are affected in only 10–15 % of cases [17, 18]. Clinical phenotype observed among males include young age, focal lesions and acute vascular complications, such as artery dissection [17, 19].

As mentioned, the symptoms of acute renal infarction are unspecific and vary from case to case. During both renal infarction episodes our patient had hypertension and acute kidney injury. Due to the decrease in renal artery diameter or total artery occlusion, a rapid drop in renal perfusion pressure occurs. As a result, natrium sieving at the glomerulus is reduced which leads to renin release and through the activation of renin-aldosterone-system to increase in blood pressure. Moreover, acute drop in renal perfusion leads to acute kidney injury, which is a common symptom of renal infarction, present in almost 50% of cases [2]. Notably, no other typical findings such as LDH elevation as an indicator for acute ischemia were observed. Initial ultrasound showed post-stenotic waveforms within the renal tissue, described with prolonged acceleration time and low resistive indices, and no blood flow via main supplying artery. Further imaging, CT-angiography respectively, has shown that the aorto-renal bypass was occluded. In addition, discrete perfusion of the kidney, through the highly developed collateral circulation was observed. Apparently, collateral blood flow was not enough to produce sufficient pressure needed for diuresis. Though, it was enough to sustain sufficient oxygenation of renal tissue. This was proven, as renal function recovered, and blood pressure normalized promptly after aorto-renal bypass two weeks after admission of the patient. This supports the role of collateral perfusion in maintaining tissue viability and suggesting predictive value for treatment success.

While many acute renal infarctions are managed conservatively [20], main artery occlusions often require intervention. Minimally invasive approaches are preferred [21]. Thrombolysis, thrombectomy, ballon angioplasty with or without the stent placement can be offered. Such treatment options are related to high success rates and low complication risk compared to surgical approach [21, 22]. The use of minimally invasive procedures in the presence of aortic-renal bypass occlusion are limited to case reports [23]. Our patient previously failed thrombectomy, necessitating surgical bypass. According to studies on patients who underwent renal revascularization surgeries, graft patency ranged from 88–97 % at 3–5 years [21, 24]. Vein grafts have slightly lower patency, possibly due to venous valves causing acute thrombosis. They are also prone to stenosis and aneurysm formation [22, 23]. Even though in our case the venous graft displayed long patency, the presence of extremely well-developed collateral vasculature suggests chronic ischemia. The long graft history and the delayed diagnosis of acute renal infarction were critical factors that influenced the decision against minimally invasive procedure in this case.

There is data that the development of collateral vasculature is associated with the severity of atherosclerotic renal artery stenosis (RAS) [25]. However, this process is better described in the context of ischemic heart disease and peripheral artery disease [26, 27]. Most often collaterals branch out from the aorta, as well as intercostal, hypogastric, ovarian and testicular arteries [28]. The exact mechanisms leading to arteriogenesis (expansion of pre-existing vessels) and angiogenesis (sprouting of arteries from existing vessels) are not well understood. Factors, such as renin-angiotensin-aldosterone system blockade, exercise and immunological growth factors have been mentioned [29]. Though variable, the ability of these collateral blood vessels to compensate organ perfusion can be as high as 40 % [28]. Moreover, it is postulated that in cases with highly developed collateral vasculature a revascularization procedure preserves renal function as well as renal blood flow [25].

## Conclusion

Our case highlights the compensatory role of collateral vasculature in acute renal infarction. Despite the complete occlusion of the bypass, collateral vasculature sustained renal tissue viability for two weeks. These findings suggest that collateral development may occur beyond the context of atherosclerotic artery disease and should be carefully evaluated during the work-up of acute renal infarction cases, as it may extend the therapeutic window for successful intervention.

## Data Availability

Data sharing is not applicable to this article as no datasets were generated or analyzed during the preparation of this case report.
